# Novel recruitment strategy to enrich for *LRRK2* mutation carriers

**DOI:** 10.1002/mgg3.151

**Published:** 2015-05-06

**Authors:** Tatiana Foroud, Danielle Smith, Jacqueline Jackson, Jennifer Verbrugge, Cheryl Halter, Leah Wetherill, Katherine Sims, Winnie Xin, Vanessa Arnedo, Shirley Lasch, Kenneth Marek, the Parkinson's Progression Markers Initiativea

**Affiliations:** 1 Department of Medical and Molecular Genetics, Indiana University School of MedicineIndianapolis, Indiana, 46202; 2 Department of Neurology, Center for Human Genetic Research, Massachusetts General HospitalBoston, Massachusetts, 02114; 3 Michael J. Fox FoundationNew York City, New York; 4 Institute for Neurodegenerative DisordersNew Haven, Connecticut, 06510

**Keywords:** Parkinson disease, LRRK2, genetic testing

## Abstract

The *LRRK2* G2019S mutation is found at higher frequency among Parkinson disease (PD) patients of Ashkenazi Jewish (AJ) ancestry. This study was designed to test whether an internet-based approach could be an effective approach to screen and identify mutation carriers. Individuals with and without PD of AJ ancestry were recruited and consented through an internet-based study website. An algorithm was applied to a series of screening questions to identify individuals at increased risk to carry the LRRK2 G2019S mutation. About 1000 individuals completed the initial screening. Around 741 qualified for mutation testing and 650 were tested. Seventy-two individuals carried at least one *LRRK2* G2019S mutation; 38 with PD (12.5%) and 34 without (10.1%). Among the AJ PD participants, each affected first-degree relative increased the likelihood the individual was LRRK2+ [OR = 4.7; 95% confidence interval = (2.4–9.0)]. The same was not observed among the unaffected AJ subjects (*P* = 0.11). An internet-based approach successfully screened large numbers of individuals to identify those with risk factors increasing the likelihood that they carried a LRRK2 G2019S mutation. A similar approach could be implemented in other disorders to identify individuals for clinical trials, biomarker analyses and other types of research studies.

## Introduction

Parkinson disease (PD) is the second most frequent neurodegenerative disorder among the elderly. Familial forms of PD comprise only 10% of cases (Elbaz et al. 1999) but have yielded important pathophysiological insights through the identification of novel genes contributing to PD susceptibility. Mutations in five genes result in Mendelian forms of PD. Two genes, leucine-rich repeat kinase 2 (*LRRK*2) and synuclein, alpha (non-A4 component of amyloid precursor) (*SNCA*), are mutated in autosomal dominant forms of PD. Three genes, parkin RBR E3 ubiquitin protein ligase (*PARK2*), DJ1 (*PARK7*) and PTEN-induced putative kinase 1 (*PINK1*) are mutated in autosomal recessive forms of PD.

The *LRRK2* G2019S mutation is the most common, single cause of PD that has been identified to date. This mutation is found at higher frequency in populations from Northern Africa as well as individuals of Ashkenazi (Eastern European) ancestry. Among PD patients of Ashkenazi Jewish (AJ) ancestry, screening studies have found the frequency of the *LRRK2* G2019S mutation to be as high as 15–20% (Orr-Urtreger et al. 2007; Ozelius et al. 2006). The penetrance of this mutation has been reported to be reduced (Goldwurm et al. 2007; Healy et al. 2008; Hentati et al. 2014; Hulihan et al. 2008; Latourelle et al. 2008; Troiano et al. 2010), with the most recent estimates suggesting that the penetrance may be as low as 30% at age 80 years (Marder et al. 2014).

The Parkinson’s Progression Markers Initiative (PPMI) is an observational clinical study using advanced imaging, biologic sampling, and clinical and behavioral assessments to identify biomarkers of PD progression. The focus of PPMI is to identify individuals at greatest risk of developing PD in order to detect biomarkers of phenoconversion and early disease progression. One of the challenges to identify these biomarkers is the identification of individuals at increased risk of PD in whom biomarkers could be monitored. One approach to identify individuals at increased risk of PD is to enrich for subjects more likely to have genetic risk factors, such as the *LRRK2* G2019S mutation. Even with the availability of this risk factor, it is still financially costly and time consuming to screen large numbers of individuals to identify those that carry this mutation.

The PPMI study has developed an innovative approach to screen large numbers of individuals from enriched populations. In this study, we focused on the recruitment of individuals of AJ ancestry and then further enriched the sample by asking about a family history of PD. To reduce costs, all individuals completed their screening and consenting process online and provided a saliva sample through the mail for genetic testing. As we describe in this report, our strategy has proven extremely successful and led to the identification of a large number of individuals, both with and without a diagnosis of PD, who carry a *LRRK2* G2019S mutation. These individuals have now been invited to participate in the biomarker aspect of the PPMI study.

## Methods

### Outreach and participant screening

**Figure 1:**
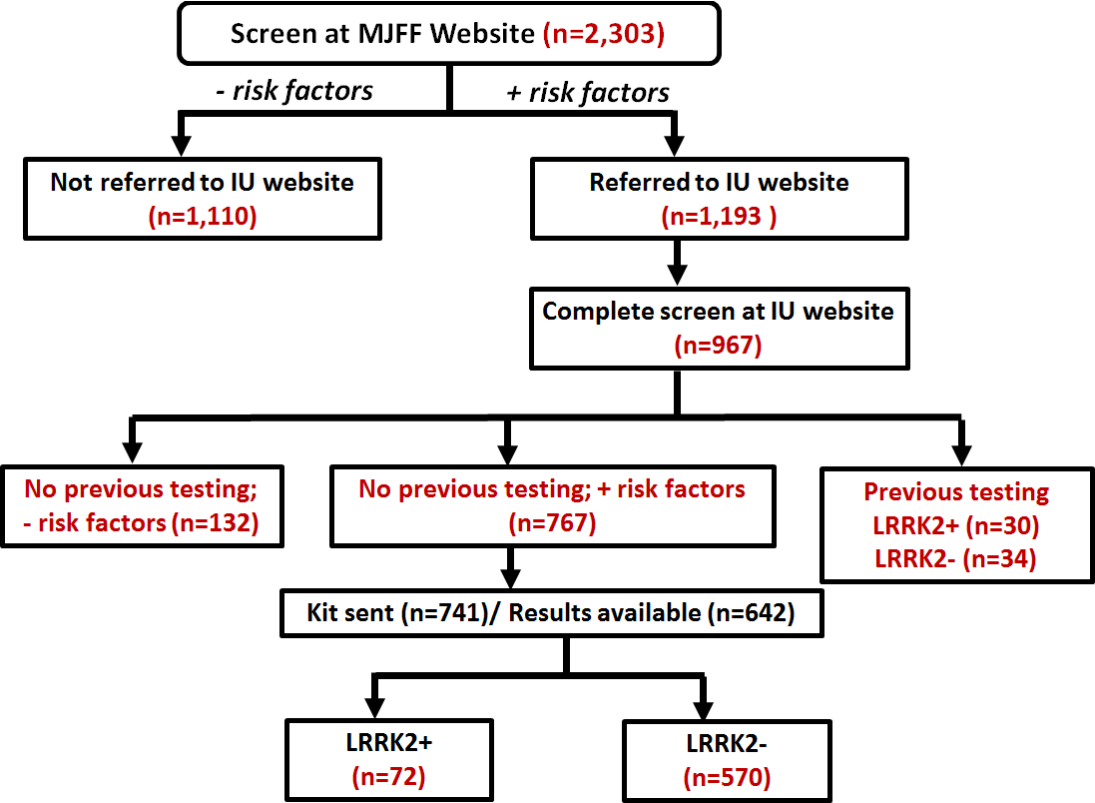
Study overview.

The Michael J. Fox Foundation prepared recruitment material that highlighted the higher frequency of the *LRRK2* G2019S mutation in the AJ population. The recruitment materials were widely distributed through print, e-mail and in-person campaigns to individuals of AJ ancestry. In addition, recruitment materials were also distributed broadly by the Michael J. Fox Foundation to individuals interested in PD research. Interested individuals were directed to the Michael J. Fox Foundation PPMI website for an initial screening to determine if they had risk factors that indicated an increased risk of a *LRRK2* mutation (Fig.1). Individuals who met the initial criteria were then provided a link which directed them to a website at Indiana University.

The website at Indiana University presented a fact sheet about *LRRK2* and PD. Individuals could then choose to continue to the online Informed Consent document. The Informed Consent document described the study, provided additional information about *LRRK2* and PD, and provided the risks and benefits of study participation. Individuals were then asked if they had any questions about the study. If they indicated that they had questions, they were asked to provide their name and contact information so that study personnel could contact them to answer their questions. Individuals who indicated that they had questions were not given the option to agree to the electronic consent or continue with the screening process until after they had been contacted by study personnel. If the individual did not have any questions, they indicated that they agreed to the terms of the Informed Consent form by providing their name. They were then asked to provide their contact information (mailing address, e-mail address and telephone number).

**Figure 2:**
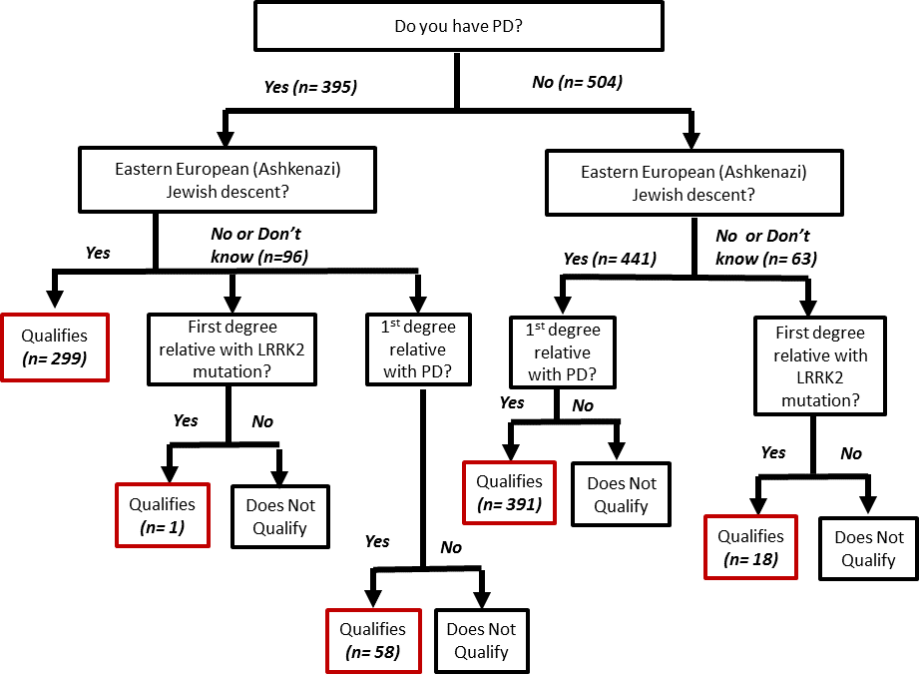
Screening algorithm.

After providing their contact information, the participant was directed to an online study case report form (Fig.1). Participants were asked a series of questions to determine whether they had risk factors that would increase the likelihood that they carried a *LRRK2* G2019S or R1441G mutation (Table1). Questions included whether or not they had PD, if they were of AJ ancestry, and whether or not they had a first-degree relative with PD. An algorithm based on the participant’s responses determined the individual’s eligibility to participate in the next phase of the study (Fig.2). Individuals with a diagnosis of PD had to report AJ ancestry and/or a first-degree relative with PD to qualify to receive genetic testing. To qualify for genetic testing, participants who did not have a diagnosis of PD were required to be of AJ ancestry and to also have a first-degree relative with PD. Any individual, regardless of PD status, who reported a first-degree relative with a *LRRK2* mutation also qualified for genetic testing. A map of the US PPMI sites was displayed and participants were reminded that participation in the full PPMI study would require that they visit a PPMI site several times over the upcoming years. They were then asked to select the PPMI site that would be most convenient for them to go to for study visits. At the end of the screening questions and site selection, the individual received a message indicating whether they were eligible to participate in the genetic screening phase of the study.

**Table 1:** Results for the screening questions for 642 individuals with results available for *LRRK2* G2019S genetic testing

Question	Response	Number of responses (%)	No. *LRRK2* G2019S+ (%) (*n* = 72)	No. *LRRK2* G2019S− (%) (*n* = 570)
1. Do you have Parkinson disease?	Yes	305 (47.5)	38 (52.8)	267 (46.8)
	No	337 (52.5)	34 (47.2)	303 (53.2)
2. Have you previously been tested for a possible *LRRK2* gene mutation? (no report found to be available)	Yes	13 (2.0)	5 (6.9)	8 (1.4)
	No	582 (90.7)	60 (83.3)	522 (91.6)
	Do not know	47 (7.3)	7 (9.7)	40 (7.0)
3. Are you of Eastern European (Ashkenazi) Jewish descent?	Yes	579 (90.2)	67 (93.1)	512 (89.8)
	No	43 (6.7)	3 (4.2)	40 (7.0)
	Do not know	20 (3.1)	2 (2.3)	18 (3.2)
4. Are you of Basque (Northern Spain) descent? (newer question, 356 responding)	Yes	3 (0.8)	0 (0.0)	3 (0.5)
	No	335 (94.1)	35 (48.6)	300 (52.6)
	Do not know	18 (5.1)	4 (5.6)	14 (2.5)
5. Do any of the following also have PD?	Father	222 (34.6)	26 (36.1)	196 (34.4)
	Mother	161 (25.1)	25 (34.7)	136 (23.9)
	Brother	50 (7.8)	5 (6.9)	45 (7.9)
	Sister	35 (5.5)	5 (6.9)	30 (5.3)
	Children	9 (1.4)	1 (1.4)	8 (1.4)
6. Do you have a first-degree relative (father, mother, full sibling, child) with a positive *LRRK2* gene test?	Yes	57 (8.9)	12 (16.7)	44 (7.7)
	No	143 (22.3)	14 (19.4)	129 (22.6)
	Do not know	443 (69.0)	46 (63.9)	397 (69.6)

Individuals who responded that they had already undergone genetic testing were informed that they would be contacted and asked to send a copy of their test results to the study coordinator for verification. If a positive test result was verified, the individual was offered genetic counseling and then referred directly to a PPMI site to learn more about the PPMI study.

### Sample collection and genetic testing

After completing the online screening, each participant was contacted to review their responses and confirm that they wished to receive a saliva collection kit from which DNA would be obtained. Once confirmed, an Oragene DISCOVER (OGR-500) kit (DNA Genotek Inc., Kanata, Ontario, Canada) was shipped to them by mail along with directions for saliva collection. The sample was then shipped back to Indiana University. Samples received at Indiana University were sent for genetic testing at Massachusetts General Hospital. Samples were tested for the *LRRK2* G2019S or *LRRK2* R144G mutation based on the participant’s response to the screening questions.

Genomic DNA was extracted from patients’ peripheral leukocytes (blood) or buccal epithelial cells (saliva) using QIAcube DNA Purification System by QIAGEN (Valencia, CA). The standard protocols provided by the manufacturer were followed. Patient DNA was PCR amplified using appropriate primer pairs depending on the specific mutation screened (*LRRK2* exon 31 c.44321C>A, p.Arg1441Gly; *LRRK2* exon 41 c.6055G>A, p.Gly2019Ser). The primers were manufactured by Invitrogen (Life Technologies, Grand Island, NY) and each primer pair was used to amplify its corresponding exon and the adjacent intronic sequences. Positive mutations were identified by comparison of bidirectional sequence data against reference sequence.

Genetic testing results were sent by Massachusetts General Hospital to Indiana University. An appointment was made for the genetic counselor to review the genetic testing results with the participant by telephone. Following counseling, the participant was provided with a written summary of the genetic testing results and counseling session.

### Statistical analysis

Logistic regression was performed to identify those variables that predicted whether an individual was *LRRK2* positive (*LRRK2+*) or *LRRK2* negative (*LRRK2−*). Due to the differential screening criteria for affected and unaffected individuals, analyses were performed in each group separately. Analyses performed in the two subject groups of AJ descent (PD and unaffected) included as potential predictors age, gender and the number of first-degree relatives with PD. Secondary analyses were performed testing for the effect of each type of relative with PD (father, mother, brother, sister, offspring).

## Results

The Michael J. Fox Foundation and Indiana University websites were activated on February 25, 2014. All results are reported for individuals who completed their consent at the Indiana University site by September 1, 2014. Missing data or kits are truncated as of October 20, 2014. A total of 2303 individuals completed the initial survey at the Michael J. Fox Foundation website and 1193 individuals qualified to be directed to the Indiana University website for further screening (see Fig.1).

A total of 967 individuals completed the consent process at the Indiana University website. Responses to the screening questions are shown in Table1. Eighty-one individuals reported having previous genetic testing which they believed indicated they carried a *LRRK2* mutation. Sixty-four of these individuals were able to provide a copy of their testing report to Indiana University for review. Upon review of the genetic testing results, slightly more than half of this group (53.1%) did not carry a *LRRK2* G2019S mutation. The majority of these results were from testing performed by 23andMe. Of the remaining 17 individuals who indicated previous testing, 4 could not be reached by the study coordinator and 13 could not produce a copy of their results. For those 13 unable to provide their test results, responses to the screening questions were used to determine eligibility for a genetic testing.

Table2 summarizes basic demographic information for the 899 individuals who completed the screening questions at the Indiana University website to determine eligibility for genetic testing. Of these 899 individuals, 395 reported a diagnosis of PD and 504 did not. The study coordinator attempted to contact all individuals to verify their screening responses. Of note, 14 individuals who had reported AJ ancestry during the survey reported that they were not actually of AJ descent during these calls, and eight individuals who had reported a relative with a known *LRRK2* mutation could not confirm that such testing had been performed.

**Table 2:** Summary and demographic information for individuals who completed initial screening at the Indiana University website by January 9, 2014

	Number	%	% M	% PD
Consented through WRI site, no previous testing	899	100	42	44
Qualified for genetic testing	767	85	45	47
Qualified and confirmed through WRI	741	82	45	47
Did not qualify through WRI	132	15	26	29
Unable to contact to confirm further participation	15	2	20	47
Declined further participation in study	11	1	46	27
Kits sent	741^1^	100	45	47
Kits not returned	91	12	33	45
Kits with *LRRK2* G2019S results from MGH	642	87	47	48
*LRRK2* G2019S	642	100	47	48
*LRRK2* G2019S-	570	89	47	47
*LRRK2* G2019S+	72	11	44	53
Consented through WRI site, with previous testing report available	64	100	41	66
*LRRK2* G2019S-	34	53	35	79
*LRRK2* G2019S+	30	47	47	50

1 Genetic testing results have not been received for 5 individuals, and there were 3 sample failures.

An algorithm was applied to determine eligibility for genetic testing (Fig.2). Individuals with a diagnosis of PD had to report at least one of the following to qualify: (1) AJ ancestry (*n* = 299); (2) first-degree relative with PD (*n* = 58); or (3) first-degree relative with a *LRRK2* mutation (*n* = 1). A total of 358 individuals with PD met these requirements for a saliva kit. Individuals who did not have a diagnosis of PD were required to meet one of the two following criteria: (1) have a first-degree relative with a *LRRK2* mutation (*n* = 18); or (2) be of AJ ancestry and have a first-degree relative with PD (*n* = 391). About 409 unaffected individuals met these requirements for a saliva kit used for genetic testing.

Only a small number (*n* = 26) who qualified to receive a saliva kit elected not to do so or could not be reached by the study coordinator to confirm participation. Among the 741 individuals sent a saliva kit, 650 (87.7%) returned the filled kit to Indiana University. The vast majority of individuals were screened for the *LRRK2* G2019S mutation (99.9%). Only five individuals qualified to be screened for the *LRRK2* R1441G mutation in addition to the *LRRK2* G2019S, and one for the *LRRK2* R1441G mutation only. None of the subjects were positive for the *LRRK2* R1441G mutation.

*LRRK2* G2019S testing results were available for a total of 642 individuals (Table1). Results for 5 individuals were still pending and 3 samples failed when tested. This group of 642 individuals included 305 with a diagnosis of PD (47.5%) and 337 who did not. The total number of individuals carrying a *LRRK2* G2019S mutation was 72 (11.2%). Among those with PD, the number carrying a *LRRK2* G2019S mutation was 38 (12.5%) and among those without a diagnosis of PD, the number was 34 (10.1%). There were two individuals who were homozygous for the *LRRK2* G2019S mutation. Both had been previously diagnosed with PD; however, only one had a family history of PD.

The family history question on the screening survey was a strong predictor of *LRRK2* mutation status. A majority (76.4%) of individuals found to have a *LRRK2* G2019S mutation had at least one relative with PD, and 9.7% reported more than one relative with PD. Among those carrying the *LRRK2* mutation, the most frequently reported relative with PD was a parent; 36.1% reported their father with PD, and 34.7% reported an affected mother. Two *LRRK2* G2019S heterozygotes (2.8%) reported both their parents had PD.

Of the individuals with *LRRK2* G2019S testing results available, there were 254 AJ individuals with PD. Although the majority did not carry a *LRRK2* mutation (*n* = 219, 86.2%), there were 35 individuals who did (13.8%). Neither age nor gender were significant predictors of *LRRK2* mutation status (*P* > 0.94). However, the number of affected relatives was a significant predictor of mutation status (Wald *χ*^2^ = 21.0, d*f* = 1, *P* < 4.9 × 10^−6^). For each additional affected first-degree relative, the odds that the study participant had a *LRRK2* mutation increased by 4.7 [95% confidence interval (CI) = (2.4–9.0)]. Secondary analyses revealed that having an affected father significantly increased the odds the participant carried a *LRRK2* mutation by 3.7 [*P* = 0.01, 95% CI = (1.4–9.8)]. If the participant’s mother was affected, the odds the participant was LRRK2+ more than doubled, with an odds ratio of 8.1 [*P* = 3.9 × 10^−5^, 95% CI = (3.0–22.1)].

Of the individuals with results available, there were 325 AJ individuals who did not report a diagnosis of PD. Of those, 293 were *LRRK2−* (90.1%), and 32 were *LRRK2+* (9.9%). None of the tested variables was a significant predictor of *LRRK2* status (gender *P* = 0.83, age at interview *P* = 0.08, number of relatives *P* = 0.11).

## Discussion

The identification of individuals who have inherited a *LRRK2* mutation is challenging. In this study, we designed an efficient approach that allowed us to screen nearly 1000 individuals to determine if they carried risk factors that increased the likelihood that they carried a *LRRK2* mutation. With this strategy, we newly identified 72 individuals who carry a *LRRK2* G2019S mutation and also confirmed the result for 30 individuals who had received previous genetic testing. Thus, in only 6 months, we identified over 100 individuals who qualified to participate in the PPMI biomarker study focused on longitudinal evaluation of individuals carrying a *LRRK2* G2019S mutation.

Previous studies have reported that approximately 15–20% of PD patients of AJ ancestry carry the *LRRK2* G2019S mutation. In this study, we found a rate of 12.5%. This is slightly lower than the previously reported rate, but still supports the higher frequency of this mutation in this population. Prior to initiating this study, we predicted that unaffected individuals of AJ ancestry who had a first-degree relative with PD would also have an elevated frequency of the G2019S mutation. We anticipated that this rate would be half that of the PD population. In this study, we found the rate to be 10.2%, slightly higher than we would have anticipated. This may be in part due to some unaffected individuals being referred to the study by a family member who learned of their *LRRK2* carrier status.

From this study, we confirmed the importance of collecting family history information prior to genetic testing for the *LRRK2* G2019S mutation. Individuals with PD were not required to have another family member with PD to qualify for genetic testing. However, the presence of each additional affected family member with a mutation increased the likelihood that an AJ individual with PD carried the LRRK2 mutation by 4.7%. Individuals who were unaffected were not eligible for genetic testing unless they had a first-degree relative with PD. We found that among this group, the number of additional affected family members was not a significant predictor of *LRRK2* status.

We did find that individuals with an affected parent were more likely to carry a LRRK2 mutation as compared with those who did not have an affected parent. Furthermore, in our study, having an affected mother was a stronger predictor of LRRK2+ status as compared with an affected father. These results appear to be consistent with an earlier study by Alcalay et al. (2013) which found that the penetrance of *LRRK2* G2019S may be higher in women than men. If female carriers of the LRRK2 mutation are more likely to be affected, we would expect to find more affected mothers than affected fathers in our sample. This finding requires further evaluation in additional samples.

Potential study participants appeared to be unsure of previous genetic testing in family members. On the initial screen, 92 individuals reported that a family member had been previously tested and found to carry a *LRRK2* mutation. When the subject was contacted by the study coordinator to confirm this information, the majority was unable to confirm that a genetic test had been performed and was positive. In these cases, if the subject still met criteria for genetic testing, they were sent a saliva kit. If they did not qualify for a genetic testing, they did not participate further in the study.

The cost of recruiting subjects through an internet-based strategy as used in this study is difficult to quantify. Study personnel developed the website and databases required for this study. A full time coordinator was available to answer questions and also spoke by telephone to each individual who qualified for genetic testing prior to the shipping of the saliva kit. This personal interaction likely increased the commitment of the study participants. Surprisingly, few reminders to return a saliva kit were required in this subject group. Anecdotally, subjects found it convenient to participate in the study without leaving their home. Thus, by coupling a convenient online participation with a telephone call with study staff, we likely ensured that those subjects who received the saliva kit were likely to also return the kit and receive study results.

The approach used in this study could be applied quite easily for the testing of other PD susceptibility genes or to other disorders. We have just initiated recruitment for another risk factor, glucocerebrosidase (*GBA*), using the same study design. There is an ongoing study that has implemented an internet-based program for AJ carrier screening for multiple genetic disorders (Grinzaid et al. 2014). There is growing interest to utilize precision genomics, which will target disease treatment based on the patient’s underlying genetic risk profile. It will be essential that trials can be initiated that recruit participants with particular risk factors or mutations. Approaches such as ours could dramatically increase the rate at which such focused patient populations could be efficiently identified.

One weakness of the study was a targeted recruitment, focused largely on individuals of AJ ancestry. Therefore, the conclusions drawn about the participation rates and the frequency of *LRRK2* G2019S mutation are not applicable to individuals of other ancestry. However, results suggest that this recruitment strategy is successful in identifying an at-risk population. This study had several other strengths. The population being recruited was highly engaged, and for the most part had access to the internet. Only a small number of individuals (<20) required the screening questionnaire to be mailed to them because they did not have internet access. As a group, this subject population was motivated to participate fully in the study. Among those who qualified for genetic testing based on their screening questions, only 11 (1.4%) did not wish to receive a saliva kit and 15 (2.0%) did not respond to the coordinator’s telephone call to confirm their continued participation. Upon receiving a saliva kit, 87.7% returned the filled saliva kit. A total of 91 individuals (12.3%) did not return their saliva kit. Given that this is a study conducted largely by mail and the internet, the return rate for the saliva kits is very high.

In summary, we used an internet-based approach to screen large numbers of individuals to identify those with risk factors increasing the likelihood that they carried a mutation contributing to PD susceptibility. This was a highly efficient approach that in only 6 months yielded over 100 individuals who carried a *LRRK2* mutation. We believe that a similar approach could be implemented in other disorders to identify individuals for clinical trials, biomarker analyses, and other types of research studies.

## Appendix

### Steering committee

Kenneth Marek, MD^1^ (Principal Investigator); Danna Jennings, MD^1^ (Site Investigator, Olfactory Core, PI); Shirley Lasch, MBA^1^; Caroline Tanner, MD, PhD^9^ (Site Investigator);Tanya Simuni, MD^3^ (Site Investigator); Christopher Coffey, PhD^4^ (Statistics Core, PI); Karl Kieburtz, MD, MPH^5^ (Clinical Core, PI); Renee Wilson, MA^5^; Werner Poewe, MD^7^ (Site Investigator); Brit Mollenhauer, MD^8^ (Site Investigator); Douglas Galasko, MD^15^; Tatiana Foroud, PhD ^16^ (Genetics Coordination Core, BioRepository PI); Todd Sherer, PhD^6^; Sohini Chowdhury^6^; Mark Frasier, PhD^6^; Catherine Kopil, PhD^6^; Vanessa Arnedo^6^.

### Study cores

*Clinical Coordination Core*: Cynthia Casaceli, MBA^5^; *Imaging Core*: John Seibyl, MD ^1^; Susan Mendick, MPH^1^; Norbert Schuff, PhD^9^; *Statistics Core*: Christopher Coffey, PhD^4^; Chelsea Caspell ^4^; Liz Uribe^4^; Eric Foster ^4^; Katherine Gloer PhD^4^; Jon Yankey MS^4^; *Bioinformatics Core*: Arthur Toga, PhD^10^ Principal Investigator), Karen Crawford, MLIS^10^; *BioRepository*: Danielle Elise Smith^16^; Paola Casalin^12^; Giulia Malferrari^12^; *Bioanalytics Core*: John Trojanowski, MD, PhD^13^ (Principal Investigator), Les Shaw, PhD^13^ (Co-Principal Investigator); *Genetics Core*: Andrew Singleton, PhD^14^, (Principal Investigator); *Genetics Coordination Core*: Cheryl Halter^16^.

### Site investigators

David Russell, MD, PhD^1^ Site; Stewart Factor, DO^17^; Penelope Hogarth, MD^18^; David Standaert, MD, PhD^19^; Robert Hauser, MD, MBA^20^; Joseph Jankovic, MD^21^; Matthew Stern, MD^13^; Lama Chahine, MD^13^; Shu-Ching HU, MD PhD^22^; Samuel Frank, MD^23^; Claudia Trenkwalder, MD^8^,; Wolfgang Oertel MD^34^; Irene Richard, MD^24^; Klaus Seppi, MD^7^; Eva Reiter, MD^7^; Holly Shill, MD ^25^; Hubert Fernandez, MD ^26^; Anwar Ahmed, MD^26^; Daniela Berg, MD ^27^; Isabel Wurster MD^27^; Zoltan Mari, MD^28^; David Brooks, MD^29^; Nicola Pavese, MD^29^; Paolo Barone, MD, PhD^30^; Stuart Isaacson, MD^31^; Alberto Espay, MD, MSc ^32^; Dominic Rowe, MD, PhD^33^; Melanie Brandabur MD^2^; James Tetrud MD^2^; Grace Liang MD^10^Karen Marder^35^; Jean-Christophe Corvol^36^; Jose Felix Martí Masso^37^; Eduardo Tolosa^38^; Jan O. Aasly^39^; Nir Giladi^40^; Leonidas Stefanis^41^.

### Coordinators

Laura Leary^1^; Cheryl Riordan^1^; Linda Rees, MPH^1^; Barbara Sommerfeld, RN, MSN^17^; Cathy Wood-Siverio, MS^17^; Alicia Portillo^18^; Art Lenahan^18^; Karen Williams^3^; Stephanie Guthrie, MSN^19^; Ashlee Rawlins^19^; Sherry Harlan^20^; Christine Hunter, RN^21^; Baochan Tran^13^; Abigail Darin^13^; Carly Linder^13^; Gretchen Todd^22^; Cathi-Ann Thomas, RN, MS^23^; Raymond James, RN^23^; Cheryl Deeley, MSN^24^; Courtney Bishop BS^24^; Fabienne Sprenger, MD^7^; Diana Willeke^8^; Sanja Obradov^25^; Jennifer Mule^26^; Nancy Monahan^26^; Katharina Gauss^27^; Kathleen Comyns^9^ Deborah Fontaine, BSN, MS, RN, GNP, MS ^15^; Christina Gigliotti^15^; Arita McCoy^28^; Becky Dunlop^28^; Bina Shah, BSc^29^; Susan Ainscough^30^; Angela James^32^; Rebecca Silverstein^31^; Kristy Espay^32^; Madelaine Ranola^33^; Helen M. Santana^35^; Nelly Ngono^36^; Elisabet Rezola^37^ (; Delores Vilas Rolan^38^; Bjorg Waro^39^; Anat Mirlman^40^; Maria Stamelou^41^.

### Industry Scientific Advisory Board

Thomas Comery, PhD^42^; Spyros Papapetropoulos, MD, PhD^42^; Bernard Ravina, MD, MSCE^43^; Igor D. Grachev, MD, PhD^44^; Jordan S. Dubow, MD^45^; Michael Ahlijanian, PhD^46^; Holly Soares, PhD^46^; Suzanne Ostrowizki, MD, PhD^47^; Paulo Fontoura, MD, PhD^47^; Alison Chalker, PhD^48^; David L. Hewitt, MD^48^; Marcel van der Brug, PhD^49^; Alastair D. Reith, PhD^50^; Peggy Taylor, ScD^51^; Jan Egebjerg, PhD^52^; Mark Minton, MD^53^; Andrew Siderowf, MD, MSCE^53^; Pierandrea Muglia, PhD^54^; Robert Umek, PhD^55^; Ana Catafau, MD, PhD^56.^

^1^Institute for Neurodegenerative Disorders, New Haven, CT; ^2^The Parkinson’s Institute, Sunnyvale, CA; ^3^Northwestern University, Chicago, IL; ^4^University of Iowa, Iowa City, IA; ^5^Clinical Trials Coordination Center, University of Rochester, Rochester, NY; ^6^The Michael J. Fox Foundation for Parkinson’s Research, New York, NY; ^7^Innsbruck Medical University, Innsbruck, Austria; ^8^Paracelsus-Elena Klinik, Kassel, Germany; ^9^University of California, San Francisco, CA; ^10^Laboratory of Neuroimaging (LONI), University of Southern California; ^11^Coriell Institute for Medical Research, Camden, NJ; ^12^BioRep, Milan, Italy; ^13^University of Pennsylvania, Philadelphia, PA; ^14^National Institute on Aging, NIH, Bethesda, MD; ^15^University of California, San Diego, CA; ^16^Indiana University, Indianapolis, IN; ^17^Emory University of Medicine, Atlanta, GA; ^18^Oregon Health and Science University, Portland, OR; ^19^University of Alabama at Birmingham, Birmingham, AL; ^20^University of South Florida, Tampa, FL; ^21^Baylor College of Medicine, Houston, TX; ^22^University of Washington, Seattle, WA; ^23^Boston University, Boston, MA; ^24^University of Rochester, Rochester, NY; ^25^Banner Research Institute, Sun City, AZ; ^26^Cleveland Clinic, Cleveland, OH; ^27^University of Tuebingen, Tuebingen, Germany; ^28^Johns Hopkins University, Baltimore, MD; ^29^Imperial College of London, London, UK; ^30^University of Salerno, Salerno, Italy; ^31^Parkinson’s Disease and Movement Disorders Center, Boca Raton, FL; ^32^University of Cincinnati, Cincinnati, OH; ^33^Macquarie University, Sydney Australia; ^34^Philipps University Marburg, Germany; ^35^Columbia Medical, New York, NY; ^36^Pitié-Salpêtrière Hospital, Paris France; ^37^University of Donostia-Service of Neurology Hospital, San Sebastian, Spain; ^38^University of Barcelona-Hospital Clinic of Barcelona, Barcelona, Spain; ^39^Norwegian University of Science and Technology, Trondheim, Norway; ^40^Tel Aviv Sourasky Medical Center, Tel Aviv, Isreal; ^41^Foundation for Biomedical research of the Academy of Athens, Athens, Greece; ^42^Pfizer, Inc., Groton, CT; ^43^Biogen Idec, Cambridge, MA; ^44^GE Healthcare, Princeton, NJ; ^45^AbbVie, Abbot Park, IL; ^46^Bristol-Myers Squibb Company; ^47^F.Hoffmann La-Roche, Basel, Switzerland; ^48^Merck & Co., North Wales, PA; ^49^Genentech, Inc., South San Francisco, CA; ^50^GlaxoSmithKline, Stevenage, United Kingdom; ^51^Covance, Dedham, MA; ^52^H. Lundbeck A/S; ^53^Avid Radiopharmaceuticals, Philadelphia, PA; ^54^UCB Pharma S.A., Brussels, Belgium; ^55^Meso Scale Discovery; ^56^Piramal Life Sciences, Berlin, Germany.
